# Urban-Suburban Differences in Public Perspectives on Digitalizing Pediatric Research: Cross-Sectional Survey Study

**DOI:** 10.2196/60324

**Published:** 2025-01-07

**Authors:** Heping Fang, Ruoling Xian, Juan Li, Yingcun Li, Enmei Liu, Yan Zhao, Yan Hu

**Affiliations:** 1 Department of Respiratory Medicine, Pediatric Research Institute, Children's Hospital of Chongqing Medical University Chongqing Key Laboratory of Child Rare Diseases in Infection and Immunity, Ministry of Education Key Laboratory of Child Development and Disorders National Clinical Research Center for Child Health and Disorders Chongqing China; 2 Department of Pediatrics, Chongqing Health Center for Women and Children Women and Children’s Hospital of Chongqing Medical University Chongqing China; 3 Department of General Surgery, Beijing Children’s Hospital Capital Medical University National Center for Children’s Health Beijing China; 4 Department of Child Health Care, Children’s Hospital of Chongqing Medical University Chongqing Key Laboratory of Child Rare Diseases in Infection and Immunity, Ministry of Education Key Laboratory of Child Development and Disorders National Clinical Research Center for Child Health and Disorders Chongqing China; 5 Children’s Health Care Center, Beijing Children’s Hospital Capital Medical University National Center for Children’s Health Beijing China

**Keywords:** pediatrics, pediatric research, digital health, public opinion, research, patient participation, urban, rural, caregiver attitudes, social media, mobile phone

## Abstract

**Background:**

Recruiting and retaining participants in pediatric research has always been challenging, particularly in healthy populations and remote areas, leading to selection bias and increased health disparities. In the digital age, medical research has been transformed by digital tools, offering new opportunities to enhance engagement in clinical research. However, public perspectives on digitalizing pediatric research and potential differences between urban and suburban areas remain unclear.

**Objective:**

This study aimed to investigate public perspectives on digitalizing pediatric research and compare differences between urban and suburban areas to help diversify participants and address health disparities.

**Methods:**

A cross-sectional web-based survey targeting caregivers of kindergarten children (aged 2-7 years) in Chongqing was conducted between June and December 2023. A total of 4231 valid questionnaires were analyzed, with 25.1% (n=1064) of the children residing in urban areas and 74.9% (n=3167) in suburban areas. Descriptive statistics and intergroup comparisons were used for data analysis.

**Results:**

Approximately 59.8% (n=2531) of the caregivers had first impressions of pediatric research, with 36.9% (n=1561) being positive and 22.9% (n=970) being negative. A total of 38.3% (n=1621) of caregivers recognized the growing popularity of digital tools, and 36.7% (n=1552) supported their use in pediatric research, but only 25.2% (n=1068) favored online-only research methods. The main concerns regarding the use of software in pediatric research were privacy issues (n=3273, 77.4%) and potential addiction (n=2457, 58.1%). Public accounts of research institutions (n=3400, 80.4%) were the most favored for online recruitment. Telephones (1916/3076, 62.3%) and social media apps (1801/3076, 58.6%) were the most popular for regular contact. Intergroup comparisons revealed that suburban caregivers had more positive first impressions of pediatric research (38.6% vs 32%; *P*<.001; adjusted odds ratio [aOR] 1.27, 95% CI 1.09-1.47) and faced fewer participation barriers: “worry about being an experimental subject” (70.9% vs 76.6%; *P*<.001; aOR 0.79, 95% CI 0.67-0.93), “pose a risk to children’s health” (58.6% vs 67.8%; *P*<.001; aOR 0.71, 95% CI 0.61-0.83), “do not have enough background information” (55.2% vs 61.6%; *P*<.001; aOR 0.78, 95% CI 0.67-0.89), and “worry about recommending other products” (48.2% vs 55%; *P*<.001; aOR 0.78, 95% CI 0.67-0.89). They also showed greater support for online-only research methods (26% vs 22.9%; *P*=.045; aOR 1.19, 95% CI 1.01-1.41) and greater openness to unofficial online recruitment sources (social media friends: 24.7% vs 18.9%; *P*<.001; aOR 1.33, 95% CI 1.11-1.59; moments on social media: 15.5% vs 11.1%; *P*<.001; aOR 1.35, 95% CI 1.09-1.67).

**Conclusions:**

In the digital age, enhancing recruitment and retention in pediatric research can be achieved by integrating both official and unofficial social media strategies, implementing a hybrid online-offline follow-up approach, and addressing privacy concerns.

## Introduction

Recruitment and retention in pediatric research have consistently posed challenges [1-3]. In the United States, pediatric randomized clinical trials are often discontinued or unpublished, with patient recruitment difficulties (37%) being the primary reason [4]. Our experiences further corroborate this trend. For instance, in a cross-sectional hospital-based survey, we recruited only 65.8% of the caregivers [5]. Similarly, in a longitudinal study on the growth of critically ill children after liver transplantation, follow-up was completed for only 68.6% of the children one year after the procedure [6]. Recruitment and retention challenges frequently prevent studies from achieving their preset objectives, resulting in inefficiency and wasted personnel and financial resources.

While traditional research has focused on children with evident illnesses, emerging concerns such as overweight, internet addiction, and emotional or behavioral issues [7-10] highlight the importance of studying seemingly healthy children [11-13]. However, recruiting and retaining healthy children for research is more challenging than for those with specific diseases, as shown by pediatric reference interval studies [14]. Additionally, children in remote areas are frequently underrepresented in research [15-17], leading to selection bias and exacerbating health disparities. In China, geographical location has been a primary longstanding contributor to child health inequity [18]. Therefore, researchers need to devise strategies to address these challenges to meet the increasing demands of pediatric research. In this context, obtaining participants’ perspectives by seeking their opinions may provide more practical solutions.

Caregivers, typically parents, are key decision makers regarding younger children’s participation in research [19]. Numerous studies have examined parental decision-making in enrolling children in pediatric research, primarily focusing on traditional research methods [20-27]. In the digital age, medical research has been significantly transformed by digital tools and the process of digitalization [28,29]. Digital medicine has created new opportunities to enhance engagement in clinical research, especially in healthy populations and remote areas, which improves health outcomes for participants [30-32]. This study investigated public perspectives on pediatric research in the digital age and examined potential differences between urban and suburban areas. The goal was to provide insights to advance the digitalization of pediatric research, which may diversify the pool of participants and address health disparities among children.

## Methods

### Study Design and Participants

This study was a cross-sectional, web-based questionnaire survey. The study targeted caregivers of kindergarten children in Chongqing, a municipality in southwestern China, with a total of 12.58 million households and 995,239 kindergarten children. Chongqing is divided into urban and suburban areas based on geographical location and development level, with 3.07 million (23.9%) households in urban areas and 9.79 million (76.1%) in suburban areas [33]. Kindergarten children are typically between 3 and 6 years old, with exceptions depending on individual circumstances [34]. The inclusion criteria for kindergarten children were (1) residency in Chongqing, (2) kindergarten attendance, (3) aged 2 to 7 years, and (4) voluntary completion of the questionnaire by the caregiver. The exclusion criteria were (1) failure to pass validation questions, (2) logical errors in responses, and (3) self-reported lack of seriousness in answering.

### Ethical Considerations

This study was approved by the Ethics Committee of the Children’s Hospital of Chongqing Medical University (Protocol #2023-236), with informed consent signatures waived. The collected data were anonymized, and no compensation was given.

### Reporting Guidelines

The CHERRIES (Checklists for Reporting Results of Internet E-Surveys) and STROBE (Strengthening the Reporting of Observational Studies in Epidemiology) guidelines were followed for reporting the results [35,36].

### Research Outcomes and Measurements

The primary outcome of this study was public perspectives on pediatric research, with differences between urban and suburban areas as the secondary outcome. These perspectives were divided into 4 key categories: “Facilitators and Barriers to Participation,” “Perspectives on Digital Medicine,” “Perspectives on Recruitment,” and “Perspectives on the Research Process.” Each category comprised single-choice and multiple-choice questions designed to collect data. In the intergroup comparison, participants were classified as urban or suburban (exposure) based on their responses to a residential address question (Multimedia Appendix 1).

### Development of the Questionnaire

The questionnaire was initially developed and refined based on the literature and clinical experience [20-27,37-39], then revised and culturally adapted by 7 pediatric doctors with diverse professional backgrounds (see Acknowledgments). The final version was converted to a web-based format using “Lediaocha,” a web-based survey platform, with 50 mandatory questions spread across 4 pages. Of these, 11 questions focused on general characteristics, while the rest investigated perspectives on pediatric research, particularly digital medicine. To ensure data reliability, one question on caregivers’ roles was repeated at the beginning and end of the questionnaire, along with a self-assessment query on response seriousness before submission. Participation was voluntary, anonymous, and accessible through WeChat (Tencent) on smartphones. Device IDs were recorded to prevent duplicate entries. When caregivers started answering, the system marked them as “in progress.” Although it was technically feasible to save “in progress” answers automatically, ethical concerns led to a decision not to do so, as these were not final submissions. The process of developing questionnaires and the methodological considerations for web-based surveys followed our team’s established practices [40].

### Determination of the Sample Size

Due to the lack of a clear sample size calculation method for multioutcome survey studies, we used the estimation method from quality-of-life studies, which suggests 5-10 questionnaires per question [41]. Therefore, with 50 questions, a minimum of 500 questionnaires were required. To mitigate potential selection bias [42], the minimum sample size was doubled to 1000. Further calculations took into account the number of households in Chongqing, with questionnaires planned to be collected at a 1:3 ratio between urban and suburban areas. As a result, a total of 4000 questionnaires were needed, with at least 1000 from urban areas and 3000 from suburban areas. Given the possibility of a high invalid response rate (approximately 50%) in public surveys [43], at least 8000 households were estimated to be needed. This sample size represented 0.4% of the entire population of kindergarten children in Chongqing, placing the sampling ratio between the national population dynamics survey (0.1%) and the sample survey (1%). Finally, the actual proportions of recruited participants from urban and suburban areas were 25.1% and 74.9%, respectively, which is consistent with the planned sample distribution.

### Survey Process

The survey was conducted between June and December 2023. Prior to the official launch, a pilot test with 50 participants was conducted to validate the survey process. A flexible survey was then conducted in urban and suburban areas over 20 days to accommodate different kindergarten schedules. The questionnaire was distributed to kindergartens and forwarded to caregivers, ensuring that recruitment focused on the target audience and minimized nontarget responses. Forwarding questionnaires by kindergarten teachers was not mandatory. Caregivers could view a short introduction and an anonymous, voluntary declaration of the study on the questionnaire homepage to avoid any sense of obligation. After the survey, researchers downloaded and saved the data from the cloud servers and then removed them from the cloud.

### Data Processing

**Figure 1:**
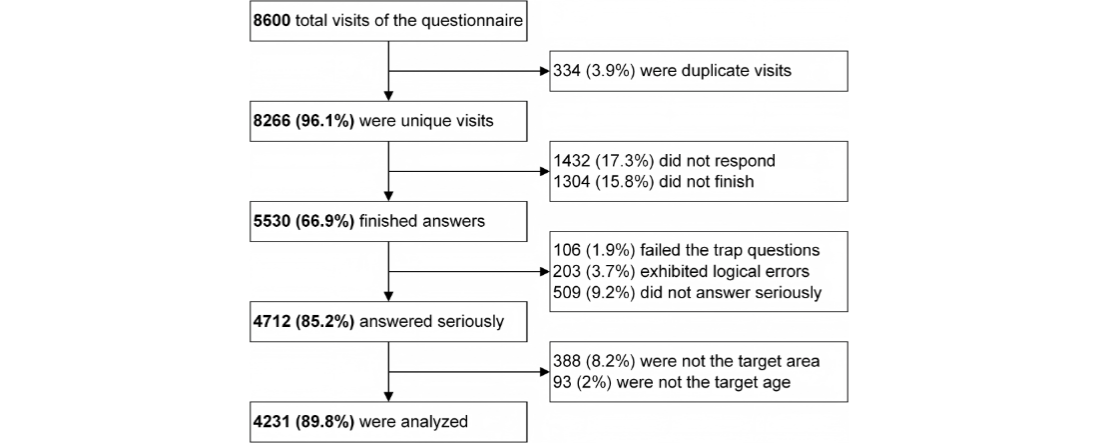
The inclusion and exclusion process.

The collected questionnaires were assessed for adherence to the inclusion and exclusion criteria. Specifically, 8600 online visits were documented during the survey, with 8266 (96.1%) identified as unique after eliminating duplicates. Among the visitors, 6834 started the questionnaire (response rate of 82.7%), and 5530 completed it (completion rate of 66.9%). After unreliable and nontarget questionnaires were excluded, 4231 questionnaires were ultimately analyzed (inclusion rate of 76.5%), representing 0.43% of the entire population of kindergarten children (Figure 1).

### Statistical Analysis

Data analysis was performed using SPSS (version 25; IBM Corp). Qualitative data are presented as frequencies (percentages), while quantitative data are presented as medians (IQRs) after tests for normality. Intergroup comparisons were conducted using chi-square and Mann-Whitney tests. Multivariate logistic regression was used to adjust for potential confounders, including child age and sex, caregiver age, role, education level, and family size, when examining the relationship between region (independent variable) and public perspectives (outcome variables). Stepwise methods were not applied (Multimedia Appendix 2). The results of the logistic regression are presented as adjusted odds ratios (aORs) with 95% CIs. A *P* value less than .05 was considered statistically significant.

## Results

### General Characteristics

Table 1 presents the general characteristics of the children and caregivers. Among the children, 1064 (25.1%) resided in urban areas, while 3167 (74.9%) children were from suburban areas. Most children (n=3774, 89.2%) had never participated in pediatric research, while only 7.7% (n=326) and 5.4% (n=230) had taken part in offline and online research, respectively. Interestingly, suburban children had a greater proportion of online research participation than urban children (6% vs 3.9%; *P*=.008). Among the caregivers, 3547 (83.8%) were mothers, 2334 (55.2%) had an education level of senior high school or below, and 2297 (54.3%) lived in households with 4 or fewer members.

**Table 1:** General characteristics of the children and caregivers.

General characteristics			Overall		Urban		Suburban		*P* value	
Age of the children (years), median (IQR)			4.8 (4.1-5.6)		4.6 (4.0-5.5)		4.9 (4.2-5.7)		<.001	
**Sex of the children, n (%)**										.44
	Male	2167 (51.2)		534 (50.2)		1633 (51.6)				
	Female	2064 (48.8)		530 (49.8)		1534 (48.4)				
Ever participated in offline pediatric research, n (%)			326 (7.7)		72 (6.8)		254 (8)		.19	
Ever participated in online pediatric research, n (%)			230 (5.4)		41 (3.9)		189 (6)		.008	
Age of the caregivers (years), median (IQR)			33 (29-36)		33 (30-36)		32 (29-36)		.81	
**Role of the caregivers, n (%)**										.005
	Mother	3547 (83.8)		921 (86.6)		2626 (82.9)				
	Father or others	684 (16.2)		143 (13.4)		541 (17.1)				
**Education levels of the caregivers, n (%)**										<.001
	Senior high school or below	2334 (55.2)		455 (42.8)		1879 (59.3)				
	College or above	1897 (44.8)		609 (57.2)		1288 (40.7)				
**Number of people in the family, n (%)**										<.001
	2-4 people	2297 (54.3)		707 (66.4)		1590 (50.2)				
	≥5 people	1934 (45.7)		357 (33.6)		1577 (49.8)				

### Facilitators and Barriers to Participation

When questioned about their perspectives on research participation, 2531 (59.8%) caregivers expressed their first impressions, with 1561 (36.9%) caregivers inclined to participate and 970 (22.9%) caregivers inclined to decline. Suburban caregivers had more positive impressions than urban caregivers (38.6% vs 32%; *P*<.001; aOR 1.27, 95% CI 1.09-1.47). Additionally, caregivers with positive impressions reported higher rates of both offline (14.2% vs 2.9%; *P*<.001) and online (9.8% vs 2.7%; *P*<.001) research participation compared to those with negative impressions.

Table 2 presents the facilitators and barriers to participation, with most facilitators reporting rates lower than the barriers. The most significant facilitator was “be beneficial for children’s health” (n=3579, 84.6%), which was more common among urban caregivers (87.5% vs 83.6%; *P*<.001; aOR 1.45, 95% CI 1.17-1.79). Interestingly, although “be suggested by close people” was deemed less important (n=635, 15%), it had a higher rate among suburban caregivers (16.2% vs 11.5%; *P*<.001; aOR 1.47, 95% CI 1.18-1.79). On the other hand, the most significant barriers were “worry about being an experimental subject” (Guinea pig concerns: n=3060, 72.3%) and “pose a risk to children’s health” (n=2577, 60.9%). These barriers reflected a general apprehension about potential health risks. Furthermore, suburban caregivers had lower reporting rates for most barriers than urban caregivers, including “worry about being an experimental subject” (70.9% vs 76.6%; *P*<.001; aOR 0.79, 95% CI 0.67-0.93), “pose a risk to children’s health” (58.6% vs 67.8%; *P*<.001; aOR 0.71, 95% CI 0.61-0.83), “do not have enough background information” (55.2% vs 61.6%; *P*<.001; aOR 0.78, 95% CI 0.67-0.89), and “worry about recommending other products” (48.2% vs 55%; *P*<.001; aOR 0.78, 95% CI 0.67-0.89).

**Table 2:** Facilitators and barriers to pediatric research participation.

Questions about facilitators and barriers		Overall, n (%)	Urban, n (%)	Suburban, n (%)	*P* value
**Facilitators**					
	Be beneficial for children’s health	3579 (84.6)	931 (87.5)	2648 (83.6)	<.001^a^
	Trust the hospital and researchers	1454 (34.4)	371 (34.9)	1083 (34.2)	.69
	Acknowledge the importance of research	1409 (33.3)	358 (33.6)	1051 (33.2)	.78
	Increase contact with doctors	1189 (28.1)	295 (27.7)	894 (28.1)	.75
	Have small burden on the participants	1139 (26.9)	323 (30.4)	816 (25.8)	.003^a^
	Be driven by altruism	1044 (24.7)	261 (24.5)	783 (24.7)	.90
	Be suggested by close people	635 (15)	122 (11.5)	513 (16.2)	<.001^a^
	Have economic subsidies	390 (9.2)	100 (9.4)	290 (9.2)	.81
**Barriers**					
	Worry about being an experimental subject	3060 (72.3)	815 (76.6)	2245 (70.9)	<.001^a^
	Pose a risk to children’s health	2577 (60.9)	721 (67.8)	1856 (58.6)	<.001^a^
	Do not have enough background information	2404 (56.8)	655 (61.6)	1749 (55.2)	<.001^a^
	Worry about personal privacy	2153 (50.9)	574 (53.9)	1579 (49.9)	.02
	Worry about recommending other products	2112 (49.9)	585 (55)	1527 (48.2)	<.001^a^
	Refuse procedures like venipuncture	1721 (40.7)	468 (44)	1253 (39.6)	.01
	Cause disagreements among family members	1106 (26.1)	294 (27.6)	812 (25.6)	.20
	Affect the purchase of medical insurance	803 (19)	201 (18.9)	602 (19)	.93

^a^Confirmed by multivariate logistic regression that adjusted general characteristics (Multimedia Appendix 2).

### Perspectives on Digital Medicine

When questioned about digital medicine (Table 3), 36.4% (n=1542) of caregivers reported following online health-related accounts, while 38.4% (n=1626) had used online information for health-related decisions. Additionally, 38.3% (n=1621) and 36.7% (n=1552) of caregivers recognized the increasing popularity of digital tools in future health care practices and supported their integration into pediatric research. Nearly half of caregivers (n=2073, 49%) were willing to collect data daily using digital tools, and 61.7% (n=2610) supported the use of gamification in pediatric research. However, only 25.2% (n=1068) of caregivers supported online-only research methods, with slightly more support from suburban caregivers (26% vs 22.9%; *P*=.045; aOR 1.19, 95% CI 1.01-1.41). When asked about concerns regarding smartphone data collection software, privacy was the main issue (n=3273, 77.4%), followed by concerns about potential software addiction (n=2457, 58.1%). Multivariate logistic regression revealed privacy concerns as the barrier to supporting digital tools in pediatric research (aOR 0.61, 95% CI 0.52-0.71).

**Table 3:** Perspectives on digital medicine.

Questions about digital medicine			Overall, n (%)		Urban, n (%)		Suburban, n (%)		*P* value
Have followed online health-related accounts			1542 (36.4)		410 (38.5)		1132 (35.7)		.10
Can use online information to make health-related decisions			1626 (38.4)		327 (30.7)		1299 (41)		<.001^a^
Knowing that digital tools will become popular in future health care practices			1621 (38.3)		411 (38.6)		1210 (38.2)		.81
Support digital tools in pediatric research			1552 (36.7)		372 (35)		1180 (37.3)		.18
Support gamification of pediatric research			2610 (61.7)		680 (63.9)		1930 (60.9)		.09
Support online-only research methods			1068 (25.2)		244 (22.9)		824 (26)		.045^a^
Be willing to use digital tools daily to collect data			2073 (49)		484 (45.5)		1589 (50.2)		.008
**Concerns about using smartphone software in research**									
	Personal privacy issues	3273 (77.4)		855 (80.4)		2418 (76.3)		.007^a^	
	Possible addiction of the tools	2457 (58.1)		606 (57)		1851 (58.4)		.39	
	Transparency of data	2159 (51)		564 (53)		1595 (50.4)		.14	
	Health impacts of the tools (such as radiation)	1830 (43.3)		439 (41.3)		1391 (43.9)		.13	
	Possible expenses	1465 (34.6)		367 (34.5)		1098 (34.7)		.92	
	Ease of use	1279 (30.1)		320 (30.1)		959 (30.3)		.90	

^a^Confirmed by multivariate logistic regression that adjusted general characteristics (Multimedia Appendix 2).

### Perspectives on Recruitment

Regarding recruitment (Table 4), 39.4% (n=1666) and 39.5% (n=1670) of caregivers expressed interest in reviewing recruitment information presented offline and online, respectively. Only 930 (22%) caregivers reported they would check both online and offline recruitment information. Notably, a greater proportion of suburban caregivers preferred checking offline recruitment information (41.2% vs 34%; *P*<.001; aOR 1.28, 95% CI 1.11-1.49). Additionally, 42.9% (n=1813) of caregivers expressed doubts about the reliability of online recruitment, while 58.1% (n=2457) doubted that research participation through online recruitment would lead to different treatments compared to offline methods.

When questioned about preferences, caregivers indicated that doctors with senior titles (n=2157, 51%) and specific researchers (n=2053, 48.5%) were the preferred recruiters, together accounting for 70.8% (n=2996) of caregivers. Research institution public accounts (n=3400, 80.4%) were the most favored source of online recruitment, with higher preferences among urban caregivers (85.9% vs 78.5%; *P*<.001; aOR 1.62, 95% CI 1.33-1.97). Although social media friends (n=984, 23.3%) and moments (n=608, 14.4%) were less popular sources for online recruitment, they were more frequently reported by suburban caregivers (friends: 24.7% vs 18.9%; *P*<.001; aOR 1.33, 95% CI 1.11-1.59; moments: 15.5% vs 11.1%; *P*<.001; aOR 1.35, 95% CI 1.09-1.67). Finally, the majority of caregivers (n=2963, 70%) preferred cartoon-style recruitment advertisements.

**Table 4:** Perspectives on recruitment.

Questions about recruitment		Overall, n (%)	Urban, n (%)	Suburban, n (%)	*P* value
Know that informed consent is required before beginning		3549 (83.9)	900 (84.6)	2649 (83.6)	.47
Be willing to review offline recruitment information		1666 (39.4)	362 (34)	1304 (41.2)	<.001^a^
Be willing to review online recruitment information		1670 (39.5)	408 (38.3)	1262 (39.8)	.39
Doubt the reliability of online recruitment information		1813 (42.9)	474 (44.5)	1339 (42.3)	.20
Believe that the treatment between online and offline recruitment is different		2457 (58.1)	603 (56.7)	1854 (58.5)	.29
**Preferred role of recruiter**					
	Doctors (senior title)	2157 (51)	549 (51.6)	1608 (50.8)	.64
	Specific researchers	2053 (48.5)	552 (51.9)	1501 (47.4)	.01
	Medical students	786 (18.6)	188 (17.7)	598 (18.9)	.38
	Doctors (ordinary title)	459 (10.8)	130 (12.2)	329 (10.4)	.10
	Nurses	294 (6.9)	75 (7)	219 (6.9)	.88
**Preferred source of online recruitment information**					
	Public accounts of research institutions	3400 (80.4)	914 (85.9)	2486 (78.5)	<.001^a^
	Friends on social media	984 (23.3)	201 (18.9)	783 (24.7)	<.001^a^
	Moments on social media	608 (14.4)	118 (11.1)	490 (15.5)	<.001^a^
Prefer cartoon version of the recruitment information		2963 (70)	752 (70.7)	2211 (69.8)	.60

^a^Confirmed by multivariate logistic regression that adjusted general characteristics (Multimedia Appendix 2).

### Perspectives on the Research Process

When questioned about the research process (Table 5), 1445 (34.2%) caregivers reported that they knew they could withdraw from the study, and 79.8% (n=1153) of them would consult with the research team before doing so. Regarding follow-up, 3076 (72.7%) caregivers reported that regular contact was necessary. Telephones (n=1916, 62.3%) were the preferred method, followed by social media apps (n=1801, 58.6%). Social media apps were more common among urban participants (64.7% vs 56.4%; *P*<.001; aOR 1.39, 95% CI 1.17-1.65). For research feedback, 4017 (94.9%) caregivers expressed willingness to receive it, with social media apps (n=2624, 65.3%) being the preferred method, followed by email (n=2535, 63.1%). Additionally, we questioned at the beginning and end of the questionnaire whether caregivers were willing to provide phone numbers in anonymous surveys; a total of 3065 (72.4%) caregivers expressed willingness in offline surveys, a rate significantly higher than that in online surveys (72.4% vs 43.4%; *P*<.001).

**Table 5:** Perspectives on the research process.

Questions about research process			Overall, n (%)		Urban, n (%)		Suburban, n (%)		*P* value
**Know that withdrawing midway is unconditionally allowed**			1445 (34.2)		366 (34.4)		1079 (34.1)		.85
	Would discuss with the research team before withdrawing	1153 (79.8)		300 (82.0)		853 (79.1)		.23	
**Believe that regular contact is necessary**			3076 (72.7)		785 (73.8)		2291 (72.3)		.36
	Preferred method (telephone)	1916 (62.3)		514 (65.5)		1402 (61.2)		.03	
	Preferred method (social media apps)	1801 (58.6)		508 (64.7)		1293 (56.4)		<.001^a^	
	Preferred method (video)	715 (23.2)		199 (25.4)		516 (22.5)		.11	
**Be willing to receive research feedback**			4017 (94.9)		1024 (96.2)		2993 (94.5)		.03^a^
	Preferred route (social media apps)	2624 (65.3)		687 (67.1)		1937 (64.7)		.17	
	Preferred route (mail)	2535 (63.1)		694 (67.8)		1841 (61.5)		<.001^a^	
	Preferred route (telephone)	2071 (51.6)		457 (44.6)		1614 (53.9)		<.001^a^	
**Be willing to provide phone number in anonymous surveys**									
	Offline surveys	3065 (72.4)		757 (71.1)		2308 (72.9)		.27	
	Online surveys	1835 (43.4)		452 (42.5)		1383 (43.7)		.50	

^a^Confirmed by multivariate logistic regression that adjusted general characteristics (Multimedia Appendix 2).

## Discussion

### Principal Findings

This study investigated public perspectives on the digitalization of pediatric research, comparing views between urban and suburban areas. The findings indicate that the current support for digital approaches in pediatric research is suboptimal but still offers valuable strategies for using digital methods to reduce urban-suburban health disparities in children. This study is relatively rare in the literature and explores innovative ways to enhance participation and equity through digital approaches. Previous studies have emphasized the importance of understanding the barriers and facilitators that parents face when considering their children’s participation in research [20-27]. As much of the research has already begun digitalization [28-32], it is beneficial to periodically pause and gather insights from participants by considering their perspectives.

### Perspectives on Pediatric Research

A previous study on neonatal clinical trials revealed that one-quarter of parents made immediate enrollment decisions [27]. Our research found that 59.8% (n=2531) of caregivers formed initial impressions of pediatric research, with 36.9% (n=1561) expressing positive inclinations and 22.9% (n=970) expressing negative inclinations. These findings emphasize the importance of recruitment strategies during initial contact. Further analysis identified health benefits as the main facilitators and risks as the primary barriers, which aligns with previous studies from other regions [20]. This suggests the results could be applicable beyond Chongqing. The lower reporting rates of facilitators than barriers may explain challenges in pediatric research. Preferences in recruitment leaned toward doctors with senior titles and specific researchers as recruiters, emphasizing the influence of authority on caregivers’ decision-making. Additionally, previous research has shown that cartoon images influence children’s decision-making [44]. This study found that 70% (n=2963) of caregivers also preferred cartoon-style recruitment materials, possibly because they convey a sense of care for children, which resonates well with caregivers [45].

### Perspectives on Digital Medicine

Public perspectives on digital medicine revealed suboptimal acceptance among caregivers. Approximately one-third of caregivers acknowledged the increasing popularity of digital tools and supported their application in pediatric research. This was reflected in a lower willingness to provide phone numbers in online surveys than in offline surveys. The transition from traditional to remote trials, particularly during the COVID-19 pandemic, has been successful [30,46], though some unsatisfactory outcomes have been reported [47,48]. Our results showed that public accounts from research institutions were the most preferred source of online recruitment, aligning with offline sources that emphasize authority. Given that 42.9% (n=1813) and 58.1% (n=2457) of caregivers doubted the reliability and consistency of online recruitment, establishing a credible public account by research institutions could enhance recruitment success on social media, a novel strategy compared to previous studies [49]. On the other hand, the most commonly mentioned methods for regular contact were telephone and social media apps. This may explain the effectiveness of the hybrid follow-up model, which encourages using both phone calls and social media [50]. However, only 25.2% (n=1068) of caregivers supported online-only research methods, highlighting the importance of face-to-face interactions. This raises important questions about the effectiveness of a fully remote model in postpandemic pediatric research. When questioned about gamification in research, 61.7% (n=2610) of caregivers were in favor. Studies on gamified medicine have shown positive outcomes [51,52], but concerns about potential risks remain [53,54]. In this study, 58.1% (n=2457) of caregivers expressed concerns about addiction. Additionally, 77.4% (n=3273) of caregivers expressed concerns about personal privacy when using smartphone software for research, which served as a barrier to supporting digital tools in pediatric research. Another advantage of digitalizing pediatric research is the availability of personalized real-time feedback, with 94.9% (n=4017) of participants willing to receive it. This meets caregivers’ needs and serves as a strategy to increase motivation [55].

### Differences Between Urban and Suburban Areas

When comparing urban and suburban areas, demographic differences were not adjusted using methods like propensity score matching, as these differences were inherent to the population. Instead, multivariate logistic regression was used to validate the results. Interestingly, suburban caregivers expressed greater interest in pediatric research and fewer barriers and concerns. These findings contradict common knowledge [15,16,56] but align with a previous study on congenital heart disease [57]. Another recent study also showed that rural caregivers had lower concerns about data privacy and security [58]. A possible explanation for suburban caregivers’ higher enthusiasm is that the suburban areas in this study may not accurately represent resource-poor regions. These caregivers demonstrate sufficient health awareness and capabilities, making them value participation in pediatric research. This is evident in their willingness to access offline recruitment information, use digital tools for data collection, and support online-only research methods. Since suburban areas usually have more kindergarten children than urban areas (eg, Chongqing [33]), they represent a valuable potential pool of potential research participants. Further analysis revealed that suburban caregivers more frequently mentioned suggestions from close people. Similarly, suburban caregivers more often cited social media friends and moments as sources of online recruitment information. While these results raise concerns about caregivers’ ability to assess the quality of online health information [59], they also highlight the potential for incorporating informal channels in recruitment strategies for suburban participants [60].

### Strengths and Limitations

This study has several strengths. First, this study examined perspectives on digital medicine, which is distinct from conventional pediatric research topics [16,20-27] and provides essential data for the digital age. Second, this study included a more diverse sample from the general public, offering a broader representation compared to studies focused on specific illnesses. Third, the sample size was larger than that in previous studies, likely providing a more population-based overview. Finally, we performed a comparative analysis between urban and suburban areas, contributing to the understanding and mitigation of health inequalities among children in different geographical locations.

Despite these strengths, the limitations require careful consideration. First, this study was conducted in Chongqing, China. It is important to consider this when generalizing the findings to other regions. Therefore, further studies across different regions or countries are recommended to expand these findings. Second, although comparisons were made between urban and suburban areas, conditions in rural areas remain unknown and require further investigation. Third, perspectives were assessed using a structured questionnaire but lacked qualitative interview data. Additionally, despite cultural adaptations and a preliminary survey, individuals may still interpret certain questions differently based on their personal experiences. Finally, although smartphones are a prominent tool in digital health, the study’s focus on them may introduce bias. However, less common digital tools may not be widely known to the general public, making it difficult to survey participants effectively about unfamiliar technologies.

### Conclusions

In the digital age, approximately one-third of caregivers support pediatric research and endorse the use of digital tools in such studies. Caregivers in suburban areas show greater enthusiasm for research and digital medicine. Integrating official and unofficial social media recruitment strategies, implementing a hybrid online-offline follow-up approach, and addressing privacy concerns could help researchers increase participant recruitment and retention rates.

## Appendix

Multimedia Appendix 1 The questionnaire (in Chinese).

Multimedia Appendix 2 Multivariate logistic regression.

## Abbreviations

aOR: adjusted odds ratio
CHERRIES: Checklists for Reporting Results of Internet E-Surveys
STROBE: Strengthening the Reporting of Observational Studies in Epidemiology

## Data Availability

The datasets generated during or analyzed during this study are available from the corresponding author upon reasonable request.

## References

[1] Wyatt TH, Li X, Fancher S, Mitoubsi AS, Pardue J (2024). Recruitment barriers of an mHealth pediatric asthma pilot study. West J Nurs Res.

[2] Niemeyer L, Mechler K, Buitelaar J, Durston S, Gooskens B, Oranje B, Banaschewski T, Dittmann RW, Häge A (2021). 'Include me if you can'—reasons for low enrollment of pediatric patients in a psychopharmacological trial. Trials.

[3] Fischer HS, Bührer C, Czernik C (2019). Hazards to avoid in future neonatal studies of nasal high-frequency oscillatory ventilation: lessons from an early terminated trial. BMC Res Notes.

[4] Pica N, Bourgeois F (2016). Discontinuation and nonpublication of randomized clinical trials conducted in children. Pediatrics.

[5] Fang H, Chen L, Li J, Ren L, Yin Y, Chen D, Yin H, Liu E, Hu Y, Luo X (2023). A web-based instrument for infantile atopic dermatitis identification (electronic version of the Modified Child Eczema Questionnaire): development and implementation. J Med Internet Res.

[6] Fang H, Li Z, Xian R, Yin Y, Wang J, Guo H, Dai X, Zhang M, Hu Y, Li Y (2023). Early life growth and developmental trajectory in children with biliary atresia undergoing primary liver transplantation. Front Pediatr.

[7] Di Cesare M, Sorić M, Bovet P, Miranda JJ, Bhutta Z, Stevens GA, Laxmaiah A, Kengne A, Bentham J (2019). The epidemiological burden of obesity in childhood: a worldwide epidemic requiring urgent action. BMC Med.

[8] Racine N, McArthur BA, Cooke JE, Eirich R, Zhu J, Madigan S (2021). Global prevalence of depressive and anxiety symptoms in children and adolescents during COVID-19: a meta-analysis. JAMA Pediatr.

[9] Christakis DA (2010). Internet addiction: a 21st century epidemic?. BMC Med.

[10] Sayal K, Prasad V, Daley D, Ford T, Coghill D (2018). ADHD in children and young people: prevalence, care pathways, and service provision. Lancet Psychiatry.

[11] Koren G (2003). Healthy children as subjects in pharmaceutical research. Theor Med Bioeth.

[12] Postma L, Luchtenberg ML, Verhagen AAE, Maeckelberghe ELM (2021). The attitudes of healthy children and researchers towards the challenges of involving children in research: an exploratory study. Res Involve Engage.

[13] Di Carlo C, Mighton C, Clausen M, Joshi E, Casalino S, Kim THM, Kowal C, Birken C, Maguire J, Bombard Y (2024). Parents' attitudes towards research involving genome sequencing of their healthy children: a qualitative study. Eur J Hum Genet.

[14] Adeli K, Higgins V, Trajcevski K, White-Al Habeeb N (2017). The Canadian laboratory initiative on pediatric reference intervals: a CALIPER white paper. Crit Rev Clin Lab Sci.

[15] Tanner A, Kim SH, Friedman DB, Foster C, Bergeron CD (2015). Barriers to medical research participation as perceived by clinical trial investigators: communicating with rural and African American communities. J Health Commun.

[16] Watson SE, Smith P, Snowden J, Vaughn V, Cottrell L, Madden CA, Kong AS, McCulloh R, Lim CS, Bledsoe M, Kowal K, McNally M, Knight L, Cowan K, Jimenez EY (2022). Facilitators and barriers to pediatric clinical trial recruitment and retention in rural and community settings: a scoping review of the literature. Clin Transl Sci.

[17] Chen Y, Sylvia S, Dill SE, Rozelle S (2022). Structural determinants of child health in rural China: the challenge of creating health equity. Int J Environ Res Public Health.

[18] Wu Q, Pan J (2020). The trends of health equity in children aged 5-12 years and its determinants in China from 1991 to 2015 [in Chinese]. J Preventive Med Inf.

[19] COMMITTEE ON BIOETHICS (2016). Informed consent in decision-making in pediatric practice. Pediatrics.

[20] Nathe JM, Oskoui TT, Weiss EM (2023). Parental views of facilitators and barriers to research participation: systematic review. Pediatrics.

[21] Engster SA, Fascetti C, Daw K, Reis EC (2019). Parent perceptions of and preferences for participation in child health research: results from a pediatric practice-based research network. J Am Board Fam Med.

[22] Tromp K, Zwaan CM, van de Vathorst S (2016). Motivations of children and their parents to participate in drug research: a systematic review. Eur J Pediatr.

[23] Sollo N, Ahlers-Schmidt CR, Davis AM, Smith TR, Dedeaux JA, McCulloh RJ (2022). Perceived barriers to clinical trials participation: a survey of pediatric caregivers. Kans J Med.

[24] Tu WJ, Li Y, Shi XD (2012). The views of Chinese parents on research participation and informed consent. J Paediatr Child Health.

[25] McKenzie PL, Siegel DH, Cullen D, Castelo-Soccio Leslie (2021). Strategies to enhance pediatric clinical trial participation: a systematic review with narrative synthesis. Pediatr Dermatol.

[26] Weiss EM, Olszewski AE, Guttmann KF, Magnus BE, Li S, Shah AR, Juul SE, Wu YW, Ahmad KA, Bendel-Stenzel E, Isaza NA, Lampland AL, Mathur AM, Rao R, Riley D, Russell DG, Salih ZNI, Torr CB, Weitkamp J, Anani UE, Chang T, Dudley J, Flibotte J, Havrilla EM, Kathen CM, O'Kane AC, Perez K, Stanley BJ, Wilfond BS, Shah SK (2021). Parental factors associated with the decision to participate in a neonatal clinical trial. JAMA Netw Open.

[27] Weiss EM, Guttmann KF, Olszewski AE, Magnus BE, Li S, Kim SY, Shah AR, Juul SE, Wu YW, Ahmad KA, Bendel-Stenzel E, Isaza NA, Lampland AL, Mathur AM, Rao R, Riley D, Russell DG, Salih ZN, Torr CB, Weitkamp J, Anani UE, Chang T, Dudley J, Flibotte J, Havrilla EM, O'Kane AC, Perez K, Stanley BJ, Shah SK, Wilfond BS (2021). Parental enrollment decision-making for a neonatal clinical trial. J Pediatr.

[28] Harmon DM, Noseworthy PA, Yao X (2023). The digitization and decentralization of clinical trials. Mayo Clin Proc.

[29] Marra C, Chen JL, Coravos A, Stern AD (2020). Quantifying the use of connected digital products in clinical research. NPJ Digital Med.

[30] Portnoy JM, Waller M, De Lurgio S, Dinakar C (2016). Telemedicine is as effective as in-person visits for patients with asthma. Ann Allergy Asthma Immunol.

[31] Alnasser Y, Proaño A, Loock C, Chuo J, Gilman RH (2024). Telemedicine and pediatric care in rural and remote areas of middle-and-low-income countries: narrative review. J Epidemiol Glob Health.

[32] Tan RKJ, Wu D, Day S, Zhao Y, Larson HJ, Sylvia S, Tang W, Tucker JD (2022). Digital approaches to enhancing community engagement in clinical trials. NPJ Digital Med.

[33] Chongqing statistical yearbook 2022. Chongqing Municipal Statistics Bureau.

[34] Kindergarten work regulations. Ministry of Education of the People's Republic of China.

[35] Eysenbach G (2004). Improving the quality of web surveys: the Checklist for Reporting Results of Internet E-Surveys (CHERRIES). J Med Internet Res.

[36] von Elm E, Altman DG, Egger M, Pocock SJ, Gøtzsche PC, Vandenbroucke JP, STROBE Initiative (2007). The Strengthening the Reporting of Observational Studies in Epidemiology (STROBE) statement: guidelines for reporting observational studies. Lancet.

[37] Jin H, Cui M, Liu J (2020). Factors affecting people's attitude toward participation in medical research: a systematic review. Curr Med Res Opin.

[38] El Achi D, Al Hakim L, Makki M, Mokaddem M, Khalil PA, Kaafarani BR, Tamim H (2020). Perception, attitude, practice and barriers towards medical research among undergraduate students. BMC Med Educ.

[39] O'Connor S, Hanlon P, O'Donnell CA, Garcia S, Glanville J, Mair FS (2016). Understanding factors affecting patient and public engagement and recruitment to digital health interventions: a systematic review of qualitative studies. BMC Med Inform Decis Making.

[40] Fang H, Lv Y, Chen L, Zhang X, Hu Y (2023). The current knowledge, attitudes, and practices of the neglected methodology of web-based questionnaires among Chinese health workers: web-based questionnaire study. J Med Internet Res.

[41] Lu Y, Fang J, Tian L, Jin H (2015). Advanced Medical Statistics.

[42] Serdar CC, Cihan M, Yücel D, Serdar MA (2021). Sample size, power and effect size revisited: simplified and practical approaches in pre-clinical, clinical and laboratory studies. Biochem Med (Zagreb).

[43] Blumenberg C, Barros AJD (2018). Response rate differences between web and alternative data collection methods for public health research: a systematic review of the literature. Int J Public Health.

[44] Tzeng YF, Gau BS (2018). Suitability of asthma education materials for school-age children: Implications for health literacy. J Clin Nurs.

[45] Levetown M, American Academy of Pediatrics Committee on Bioethics (2008). Communicating with children and families: from everyday interactions to skill in conveying distressing information. Pediatrics.

[46] Simmons LA, Phipps JE, Whipps M, Smith P, Carbajal KA, Overstreet C, McLaughlin J, De Lombaert K, Noonan D (2022). From hybrid to fully remote clinical trial amidst the COVID-19 pandemic: strategies to promote recruitment, retention, and engagement in a randomized mHealth trial. Digit Health.

[47] Zapata KA, Wang-Price SS, Fletcher TS, Johnston CE (2018). Factors influencing adherence to an app-based exercise program in adolescents with painful hyperkyphosis. Scoliosis Spinal Disord.

[48] Haynes SC, Kamerman-Kretzmer R, Khan SS, Crossen S, Lieng MK, Marcin JP, Kenyon NJ, Kim CH (2022). Telemedicine use for pediatric asthma care: a mixed methods study. J Asthma.

[49] Darko EM, Kleib M, Olson J (2022). Social media use for research participant recruitment: integrative literature review. J Med Internet Res.

[50] Qiao S, Tang L, Zhang W, Tian S, Liu M, Yang L, Ye Z (2019). Nurse-led follow-up to outpatients with cancer pain treated with opioids at home-telephone calls plus WeChat versus telephone calls only: a quasi-experimental study. Patient Prefer Adherence.

[51] Pimentel-Ponce M, Romero-Galisteo RP, Palomo-Carrión R, Pinero-Pinto E, Merchán-Baeza JA, Ruiz-Muñoz M, Oliver-Pece J, González-Sánchez M (2024). Gamification and neurological motor rehabilitation in children and adolescents: a systematic review. Neurologia (Engl Ed).

[52] Suleiman-Martos N, García-Lara RA, Membrive-Jiménez MJ, Pradas-Hernández L, Romero-Béjar JL, Dominguez-Vías G, Gómez-Urquiza JL (2022). Effect of a game-based intervention on preoperative pain and anxiety in children: a systematic review and meta-analysis. J Clin Nurs.

[53] Boendermaker WJ, Maceiras SS, Boffo M, Wiers RW (2016). Attentional bias modification with serious game elements: evaluating the shots game. JMIR Serious Games.

[54] Holtz BE, Murray K, Park T (2018). Serious games for children with chronic diseases: a systematic review. Games Health J.

[55] Wu Q, Wang X, Zhang J, Zhang Y, van Velthoven MH (2023). The effectiveness of a WeChat-based self-assessment with a tailored feedback report on improving complementary feeding and movement behaviour of children aged 6-20 months in rural China: a cluster randomized controlled trial. Lancet Reg Health West Pac.

[56] Stewart E, Milton A, Yee HF, Song MJ, Roberts A, Davenport T, Hickie I (2022). eHealth tools that assess and track health and well-being in children and young people: systematic review. J Med Internet Res.

[57] Lin WH, Chen YK, Lin SH, Cao H, Chen Q (2023). Parents' understanding and attitudes toward the use of the WeChat platform for postoperative follow-up management of children with congenital heart disease. J Cardiothorac Surg.

[58] Chow E, Virani A, Pinkney S, Abdulhussein FS, van Rooij T, Görges M, Wasserman W, Bone J, Longstaff H, Amed S (2024). Caregiver and youth characteristics that influence trust in digital health platforms in pediatric care: mixed methods study. J Med Internet Res.

[59] Li P, Chen B, Deveaux G, Luo Y, Tao W, Li W, Wen J, Zheng Y (2022). Cross-verification of COVID-19 information obtained from unofficial social media accounts and associated changes in health behaviors: web-based questionnaire study among Chinese netizens. JMIR Public Health Surveill.

[60] Vos SC, Adatorwovor R, Roberts MK, Sherman DL, Bonds D, Dunfee MN, Spring B, Schoenberg NE (2024). Community engagement through social media: a promising low-cost strategy for rural recruitment?. J Rural Health.

