# Supplementary material for: Urban-Suburban Differences in Public Perspectives on Digitalizing Pediatric Research: Cross-Sectional Survey Study
Source: J Med Internet Res. 2025 Jan 7;27:e60324. doi: 10.2196/60324 (PMC11751655; doi:10.2196/60324)
Supplement: Multimedia Appendix 1 [file jmir_v27i1e60324_app1.pdf]

家长对儿科研究及数字化的知信行调查

尊敬的家长，您好！

本问卷调查您对儿科研究及数字化的知信行，目的在于了解您的观点，改进儿科研究中可能存在的不足之处。本调查为匿名调查（自愿参与），大约占用您10分钟时间，不涉及个人隐私信息。感谢您的参与，您的参与将有助于我们更好地关注儿童健康。

1. 您是通过什么渠道进行的这项调查？【单选题】
- ○ 学校/幼儿园
- ○ 其他

2. 您所在的城市？【城市地址题】
- ○ 请选择省份
- ○ 请选择城市
- ○ 请选择区/县

3. 孩子的性别？【单选题】
- ○ 男
- ○ 女

4. 孩子的出生日期？【日期题】

5. 您是孩子的哪位亲属？【单选题】
- ○ 母亲
- ○ 父亲
- ○ 爷爷奶奶 / 外公外婆
- ○ 其他亲属

6. 您的年龄？________岁【填空题】

7. 您的最高学历（含目前在读）是？【单选题】
- ○ 初中及以下
- ○ 高中 / 中专 / 技校
- ○ 大学本科 / 专科
- ○ 硕士研究生及以上

8. 您家中有几口人居住在一起（算上孩子）？【单选题】
- ○ 2人
- ○ 3人
- ○ 4人
- ○ 5人及以上

9. 孩子的健康状况如何？【单选题】
- ○ 健康状况良好（包括偶尔感冒发烧等）
- ○ 健康状况不佳（患有较为严重的慢性疾病，请注明：______）

10. 您的孩子参加过医学研究吗？（包括问卷调查、临床试验等）【单选题】
- ○ 参加过
- ○ 没参加过
- ○ 我不确定

11. 您的孩子是否已经参加过在网络上进行招募的医学研究（包括问卷调查、临床试验等）？【单选题】
- ○ 参加过

○ 没参加过
○ 我不确定

———— 分页 ————

12. 在医院就诊时，若您的孩子受邀参与一项医学研究，您的第一反应是什么？【单选题】

○ 倾向于参加
○ 倾向于拒绝
○ 我不确定

13. 对于邀请者的身份，您更倾向于收到什么身份人员的邀请？（可多选）【多选题】

□ 高级职称医生
□ 普通职称医生
□ 护士
□ 专门的研究者
□ 医学生/研究生
□ 我不确定

14. 邀请者的穿着谈吐、精神面貌会影响您对研究的第一印象吗？【单选题】

○ 会
○ 不会
○ 我不确定

15. 您是否知道在孩子参与研究前，需要获得家长的知情同意？【单选题】

○ 知道
○ 不知道
○ 我不确定

16. 对于儿科研究招募广告的形式，您更倾向于以下哪种形式？【单选题】

○ 儿童视角的卡通版
○ 成人视角的严肃版
○ 我不确定

17. 当您看到张贴在医院或诊室的儿科研究招募信息，您会去了解吗？【单选题】

○ 会
○ 不会
○ 我不确定

18. 以下哪些是您决定让孩子参加医学研究的重要因素？（可多选）【多选题】

□ 家人、朋友建议我参与
□ 对孩子的健康有好处
□ 可以与医疗团队有更多的接触
□ 担心不参与会影响孩子接受应有的诊疗
□ 对孩子和我的负担小（如时间、经济、交通）
□ 参与研究可能有经济补偿
□ 认为这项研究很重要
□ 信任医院 / 研究人员
□ 可能对其他相同处境的孩子有好处（利他主义）
□ 我还有补充（请注明：______）

19. 以下哪些是您决定不让孩子参加医学研究的重要因素？（可多选）【多选题】

□ 可能对孩子的健康有影响

□ 可能对购买医疗保险有影响
□ 不愿意接受抽血等有创操作
□ 参与后可能带来时间、交通、经济负担
□ 担心个人 / 隐私信息泄露
□ 可能引起家庭成员之间的意见不和
□ 对这项研究不够了解
□ 不信任医院 / 研究人员
□ 担心成为试验品（小白鼠）
□ 担心推荐、使用院外的产品
□ 我还有补充（请注明：______）

20. 您认为孩子参与医学研究的决定有必要由父母双方共同决定吗？【单选题】
○ 有必要
○ 不必要
○ 无所谓

21. 假如您同意孩子参与一项医学研究，孩子的其他亲属可能会反对吗？【单选题】
○ 会
○ 不会
○ 我不确定

22. 在您的家中，孩子的哪些亲属可能会反对孩子参与医学研究的决定？（可多选）【多选题】
□ 母亲
□ 父亲
□ 爷爷奶奶 / 外公外婆
□ 其他亲属

23. 您知道您与孩子有权可以随时退出研究吗？【单选题】
○ 知道
○ 不知道
○ 我不确定

24. 当孩子已经参与一项医学研究，但您中途想要退出时，您会如何处理？【单选题】
○ 直接退出
○ 与研究人员讨论后决定
○ 我不确定

25. 若如果一项研究需要进行长时间的随访，您更愿意接受哪种方式？（可多选）【多选题】
□ 门诊随访
□ 电话随访
□ 微信（信息）随访
□ 视频随访
□ 上门随访
□ 我不确定

26. 当孩子参与一项长期随访的研究，除了规定的随访时间点外，您认为研究方是否需要与您有其他固定的联络？【单选题】
○ 需要
○ 不需要
○ 我不确定

27. 当孩子参与一项长期随访的研究，您认为研究方至少多长时间需要与您联络一次？【单

选题】
○ 每 1 周
○ 每 1 月
○ 每 2-3 月
○ 每 4-6 月
○ 需要随访时再联络
○ 我不确定

28. 当孩子参与一项长期随访的研究，研究人员与您仅通过手机联络，而没有面对面的接触，您持何种态度？【单选题】
○ 支持
○ 反对
○ 我不确定

29. 若一项研究需要长时间随访孩子的情况，什么是您愿意配合随访的原因？（选填）【填空】

30. 当孩子参与一项研究后，您是否希望收到研究结果报告？【单选题】
○ 希望
○ 不希望
○ 无所谓

31. 您希望通过什么形式接收研究结果报告？（可多选）【多选题】
□ 快递 / 邮件
□ 电话告知
□ 微信告知 / 推送

32. 当您在医院参与一项研究，研究方邀请您留下姓名、电话号码等信息，以便接收回访信息时，您会同意吗？【单选题】
○ 会
○ 不会
○ 我不确定

———— 分页 ————

33. 您了解“数字健康”吗？（泛指利用信息技术支持健康和健康相关领域，包括使用智能手机等）【单选题】
○ 了解
○ 不了解
○ 我不确定

34. 您有在互联网上关注了一些医疗机构或医生的科普账号吗？【单选题】
○ 有
○ 没有

35. 您有利用智能手机或其他智能设备进行健康管理吗？（如心率监测、睡眠监测、身体成分分析等）【单选题】
○ 有
○ 没有

36. 您是否知道未来会有越来越多的智能设备、软件等进入到儿科研究中？【单选题】
○ 知道
○ 不知道
○ 我不确定

37. 当您在网络上（朋友圈、微博等）看到医学研究招募广告，您对其可靠性的第一印象是什么？【单选题】

○ 非常可靠
○ 可靠
○ 不确定
○ 不可靠
○ 完全不可靠

38. 当网络上（朋友圈、微博等）的医学研究招募广告正好与孩子有关时，您是否会进一步了解？【单选题】

○ 会
○ 不会
○ 我不确定

39. 对于网络招募信息，您更倾向于收到什么渠道的信息？（可多选）【多选题】

□ 微信好友等
□ 微信朋友圈等
□ 研究机构官方公众号
□ 社交媒体软件等平台推广
□ 其他（请注明：______）

40. 同一项研究，您认为网络渠道招募的与医院现场招募的是否会有不同？【单选题】

○ 会
○ 不会
○ 我不确定

41. 您更倾向于收到哪种渠道的研究邀请？【单选题】

○ 网络渠道
○ 医院现场渠道
○ 都可以

42. 您对于在医学研究中使用智能手机（如使用手机扫码填写问卷，使用手机 app 上传资料等）持何种态度？【单选题】

○ 支持
○ 反对
○ 我不确定

43. 当孩子参与一项通过手机 app 收集信息的研究，您会有哪些顾虑？（可多选）【多选题】

□ 软件操作难易程度
□ 使用安全（包括辐射等）
□ 便携程度（如可能对生活学习产生不便）
□ 可能产生的费用
□ 个人信息 / 隐私安全
□ 所收集信息的透明程度
□ 担心孩子使用手机过多
□ 我还有补充（请注明：______）

44. 当孩子参与一项互联网上的研究，需要您每日花 10-20 分钟主动记录孩子的情况，您能够配合吗？【单选题】

○ 能
○ 不能

○ 我不确定

45. 当孩子参与一项互联网上的研究，需要孩子每日使用研究软件 10-20 分钟，软件自动记录孩子的情况，您能够配合吗？【单选题】

○ 能

○ 不能

○ 我不确定

46. 当孩子受邀参与一项使用便携数字设备进行的研究时（例如可穿戴式身体活动监测仪、智能手表等），您会感兴趣吗？【单选题】

○ 感兴趣

○ 不感兴趣

○ 我不确定

47. 在研究中设定小目标、友好的比赛、奖励等以提高参与者的积极性，您对此持何种态度？【单选题】

○ 支持

○ 反对

○ 我不确定

48. 当您在互联网上参与一项研究，研究方邀请您留下姓名、电话号码等信息，以便接收回访信息时，您会同意吗？【单选题】

○ 会

○ 不会

○ 我不确定

———— 分页 ————

49. 您是孩子的哪位亲属？【单选题】

○ 母亲

○ 父亲

○ 爷爷奶奶 / 外公外婆

○ 其他亲属

50. 请评估您是否认真完成此次调查？【单选题】

○ 我已认真完成全部问题

○ 出于一些原因，我没能认真完成全部问题
