# Supplementary material for: Urban-Suburban Differences in Public Perspectives on Digitalizing Pediatric Research: Cross-Sectional Survey Study
Source: J Med Internet Res. 2025 Jan 7;27:e60324. doi: 10.2196/60324 (PMC11751655; doi:10.2196/60324)
Supplement: Multimedia Appendix 2 [file jmir_v27i1e60324_app2.pdf]

First impressions

Had positive first impressions on participation	aOR	95% CI	P value
**Areas of the participants**			
Urban (reference)	-	-	-
Suburban	1.27	1.09–1.47	.002
**Age of the children, year**	0.91	0.85–0.97	.005
**Sex of the children**			
Female	1.01	0.89–1.14	.91
Male (reference)	-	-	-
**Age of the caregivers, year**	0.99	0.98–1.01	.29
**Role of the caregivers**			
Mother	0.98	0.82–1.18	.86
Father or others (reference)	-	-	-
**Education levels of the caregivers**			
Senior high school or below	0.81	0.71–0.92	.001
College or above (reference)	-	-	-
**Number of people in the family**			
Two to four people	1.01	0.89–1.15	.83
Five people or above (reference)	-	-	-

Facilitators and Barriers to Participation

"Be beneficial for children's health"	aOR	95% CI	P value
**Areas of the participants**			
Urban	1.45	1.17–1.79	.001
Suburban (reference)	-	-	-
**Age of the children, year**	1.11	1.02–1.22	.02
**Sex of the children**			
Female	1.11	0.93–1.31	.25
Male (reference)	-	-	-
**Age of the caregivers, year**	1.00	0.99–1.01	.94
**Role of the caregivers**			
Mother	1.94	1.56–2.40	< .001
Father or others (reference)	-	-	-
**Education levels of the caregivers**			
Senior high school or below	1.20	1.01–1.42	.04
College or above (reference)	-	-	-
**Number of people in the family**			
Two to four people	0.82	0.69–0.98	.03
Five people or above (reference)	-	-	-

"Have small burden on the participants"	aOR	95% CI	P value
**Areas of the participants**			
Urban	1.23	1.05–1.44	.01
Suburban (reference)	-	-	-
**Age of the children, year**	1.06	0.98–1.14	.14
**Sex of the children**			
Female	0.88	0.77–1.01	.08
Male (reference)	-	-	-
**Age of the caregivers, year**	0.99	0.98–1.00	.06
**Role of the caregivers**			
Mother	0.83	0.68–1.00	.053
Father or others (reference)	-	-	-
**Education levels of the caregivers**			
Senior high school or below	0.79	0.69–0.90	.001
College or above (reference)	-	-	-
**Number of people in the family**			
Two to four people	0.99	0.96–1.14	.87

Five people or above (reference)	-	-	-

"Be suggested by close people"	aOR	95% CI	P value
**Areas of the participants**			
Urban (reference)	-	-	-
Suburban	1.47	1.18–1.79	.001
**Age of the children, year**	1.01	0.92–1.10	.88
**Sex of the children**			
Female	0.91	0.77–1.08	.26
Male (reference)	-	-	-
**Age of the caregivers, year**	1.01	0.99–1.02	.31
**Role of the caregivers**			
Mother	0.83	0.66–1.05	.11
Father or others (reference)	-	-	-
**Education levels of the caregivers**			
Senior high school or below	1.07	0.90–1.27	.44
College or above (reference)	-	-	-
**Number of people in the family**			
Two to four people	0.98	0.83–1.17	.86
Five people or above (reference)	-	-	-

"Worry about being an experimental subject"	aOR	95% CI	P value
**Areas of the participants**			
Urban (reference)	-	-	-
Suburban	0.79	0.67–0.93	.005
**Age of the children, year**	0.94	0.87–1.01	.08
**Sex of the children**			
Female	1.14	0.99–1.30	.06
Male (reference)	-	-	-
**Age of the caregivers, year**	0.97	0.96–0.98	< .001
**Role of the caregivers**			
Mother	1.19	0.99–1.44	.07
Father or others (reference)	-	-	-
**Education levels of the caregivers**			
Senior high school or below	0.73	0.63–0.84	< .001
College or above (reference)	-	-	-
**Number of people in the family**			
Two to four people	0.86	0.75–0.98	.03
Five people or above (reference)	-	-	-

"Pose a risk to children's health"	aOR	95% CI	P value
**Areas of the participants**			
Urban (reference)	-	-	-
Suburban	0.71	0.61–0.83	< .001
**Age of the children, year**	0.99	0.93–1.06	.74
**Sex of the children**			
Female	0.98	0.87–1.11	.77
Male (reference)	-	-	-
**Age of the caregivers, year**	0.99	0.98–1.00	.12
**Role of the caregivers**			
Mother	1.03	0.86–1.23	.75
Father or others (reference)	-	-	-
**Education levels of the caregivers**			
Senior high school or below	0.70	0.61–0.79	< .001
College or above (reference)	-	-	-
**Number of people in the family**			
Two to four people	0.98	0.86–1.11	.71
Five people or above (reference)	-	-	-

“Do not have enough background information”	aOR	95% CI	P value
**Areas of the participants**			
Urban (reference)	-	-	-
Suburban	0.78	0.67–0.89	.001
**Age of the children, year**	1.00	0.94–1.07	.98
**Sex of the children**			
Female	0.98	0.87–1.11	.76
Male (reference)	-	-	-
**Age of the caregivers, year**	1.00	0.99–1.01	.57
**Role of the caregivers**			
Mother	1.37	1.15–1.64	< .001
Father or others (reference)	-	-	-
**Education levels of the caregivers**			
Senior high school or below	1.14	1.00–1.29	.04
College or above (reference)	-	-	-
**Number of people in the family**			
Two to four people	1.09	0.97–1.24	.16
Five people or above (reference)	-	-	-

“Worry about recommending other products”	aOR	95% CI	P value
**Areas of the participants**			
Urban (reference)	-	-	-
Suburban	0.78	0.67–0.89	< .001
**Age of the children, year**	1.02	0.95–1.08	.65
**Sex of the children**			
Female	1.02	0.90–1.15	.79
Male (reference)	-	-	-
**Age of the caregivers, year**	0.98	0.97–0.99	.002
**Role of the caregivers**			
Mother	1.36	1.14–1.62	.001
Father or others (reference)	-	-	-
**Education levels of the caregivers**			
Senior high school or below	0.95	0.84–1.08	.44
College or above (reference)	-	-	-
**Number of people in the family**			
Two to four people	0.97	0.86–1.10	.64
Five people or above (reference)	-	-	-

Perspectives on Digital Medicine

“Can use online information to make health-related decisions”	aOR	95% CI	P value
**Areas of the participants**			
Urban	0.63	0.55–0.74	< .001
Suburban (reference)	-	-	-
**Age of the children, year**	0.98	0.91–1.04	.45
**Sex of the children**			
Female	1.01	0.89–1.14	.89
Male (reference)	-	-	-
**Age of the caregivers, year**	1.00	0.99–1.01	.69
**Role of the caregivers**			
Mother	0.91	0.76–1.09	.31
Father or others (reference)	-	-	-
**Education levels of the caregivers**			
Senior high school or below	0.89	0.79–1.01	.08
College or above (reference)	-	-	-
**Number of people in the family**			
Two to four people	0.91	0.81–1.04	.16
Five people or above (reference)	-	-	-

"Support online-only research methods"	aOR	95% CI	P value
**Areas of the participants**			
Urban (reference)	-	-	-
Suburban	1.19	1.01–1.41	.04
**Age of the children, year**	1.01	0.94–1.09	.81
**Sex of the children**			
Female	0.95	0.83–1.09	.49
Male (reference)	-	-	-
**Age of the caregivers, year**	1.02	1.01–1.03	.005
**Role of the caregivers**			
Mother	1.00	0.82–1.22	.99
Father or others (reference)	-	-	-
**Education levels of the caregivers**			
Senior high school or below	0.90	0.78–1.03	.14
College or above (reference)	-	-	-
**Number of people in the family**			
Two to four people	1.02	0.89–1.18	.75
Five people or above (reference)	-	-	-

"Concerns about personal privacy issues"	aOR	95% CI	P value
**Areas of the participants**			
Urban	1.22	1.02–1.46	.03
Suburban (reference)	-	-	-
**Age of the children, year**	1.07	0.99–1.15	.09
**Sex of the children**			
Female	1.11	0.96–1.28	.16
Male (reference)	-	-	-
**Age of the caregivers, year**	0.98	0.96–0.99	< .001
**Role of the caregivers**			
Mother	1.45	1.20–1.77	< .001
Father or others (reference)	-	-	-
**Education levels of the caregivers**			
Senior high school or below	0.82	0.71–0.95	.008
College or above (reference)	-	-	-
**Number of people in the family**			
Two to four people	0.92	0.80–1.07	.28
Five people or above (reference)	-	-	-

Perspectives on Recruitment

"Be willing to review offline recruitment information"	aOR	95% CI	P value
**Areas of the participants**			
Urban (reference)	-	-	-
Suburban	1.28	1.11–1.49	.001
**Age of the children, year**	1.05	0.98–1.12	.18
**Sex of the children**			
Female	1.01	0.89–1.14	.88
Male (reference)	-	-	-
**Age of the caregivers, year**	1.03	1.02–1.04	< .001
**Role of the caregivers**			
Mother	1.16	0.97–1.39	.10
Father or others (reference)	-	-	-
**Education levels of the caregivers**			
Senior high school or below	1.35	1.19–1.53	< .001
College or above (reference)	-	-	-
**Number of people in the family**			
Two to four people	1.02	0.90–1.16	.72
Five people or above (reference)	-	-	-

“Prefer public accounts of research institutions”	aOR	95% CI	P value
**Areas of the participants**			
Urban	1.62	1.33–1.97	< .001
Suburban (reference)	-	-	-
**Age of the children, year**	1.08	1.00–1.17	.07
**Sex of the children**			
Female	1.02	0.87–1.18	.84
Male (reference)	-	-	-
**Age of the caregivers, year**	0.99	0.98–1.00	.10
**Role of the caregivers**			
Mother	1.09	0.88–1.35	.46
Father or others (reference)	-	-	-
**Education levels of the caregivers**			
Senior high school or below	0.70	0.60–0.82	< .001
College or above (reference)	-	-	-
**Number of people in the family**			
Two to four people	0.88	0.75–1.03	.10
Five people or above (reference)	-	-	-

“Prefer friends on social media”	aOR	95% CI	P value
**Areas of the participants**			
Urban (reference)	-	-	-
Suburban	1.33	1.11–1.59	.002
**Age of the children, year**	1.06	0.98–1.14	.14
**Sex of the children**			
Female	0.95	0.83–1.10	.50
Male (reference)	-	-	-
**Age of the caregivers, year**	1.01	0.99–1.02	.46
**Role of the caregivers**			
Mother	1.12	0.91–1.38	.29
Father or others (reference)	-	-	-
**Education levels of the caregivers**			
Senior high school or below	1.45	1.25–1.68	< .001
College or above (reference)	-	-	-
**Number of people in the family**			
Two to four people	1.05	0.90–1.21	.55
Five people or above (reference)	-	-	-

“Prefer moments on social media”	aOR	95% CI	P value
**Areas of the participants**			
Urban (reference)	-	-	-
Suburban	1.35	1.09–1.67	.007
**Age of the children, year**	1.03	0.94–1.13	.58
**Sex of the children**			
Female	0.95	0.80–1.13	.58
Male (reference)	-	-	-
**Age of the caregivers, year**	1.02	1.00–1.03	.04
**Role of the caregivers**			
Mother	1.22	0.95–1.58	.13
Father or others (reference)	-	-	-
**Education levels of the caregivers**			
Senior high school or below	1.60	1.34–1.92	< .001
College or above (reference)	-	-	-
**Number of people in the family**			
Two to four people	0.97	0.81–1.15	.71
Five people or above (reference)	-	-	-

Perspectives on Research Process

“Regular contact method (social media apps)”	aOR	95% CI	P value
**Areas of the participants**			
Urban	1.39	1.17–1.65	< .001
Suburban (reference)	-	-	-
**Age of the children, year**	0.99	0.91–1.06	.71
**Sex of the children**			
Female	1.08	0.93–1.25	.31
Male (reference)	-	-	-
**Age of the caregivers, year**	0.99	0.97–1.00	.03
**Role of the caregivers**			
Mother	1.04	0.85–1.28	.69
Father or others (reference)	-	-	-
**Education levels of the caregivers**			
Senior high school or below	0.92	0.80–1.07	.28
College or above (reference)	-	-	-
**Number of people in the family**			
Two to four people	0.99	0.86–1.15	.92
Five people or above (reference)	-	-	-

“Be willing to receive research feedback”	aOR	95% CI	P value
**Areas of the participants**			
Urban	1.49	1.04–2.13	.03
Suburban (reference)	-	-	-
**Age of the children, year**	1.24	1.07–1.43	.005
**Sex of the children**			
Female	1.02	0.78–1.35	.87
Male (reference)	-	-	-
**Age of the caregivers, year**	0.98	0.91–1.01	.13
**Role of the caregivers**			
Mother	1.75	1.24–2.48	.001
Father or others (reference)	-	-	-
**Education levels of the caregivers**			
Senior high school or below	0.84	0.63–1.12	.23
College or above (reference)	-	-	-
**Number of people in the family**			
Two to four people	0.84	0.63–1.11	.21
Five people or above (reference)	-	-	-

“Receive research feedback (mails)”	aOR	95% CI	P value
**Areas of the participants**			
Urban	1.24	1.06–1.45	.007
Suburban (reference)	-	-	-
**Age of the children, year**	0.97	0.90–1.04	.36
**Sex of the children**			
Female	0.96	0.94–1.09	.49
Male (reference)	-	-	-
**Age of the caregivers, year**	0.99	0.98–1.00	.13
**Role of the caregivers**			
Mother	0.87	0.72–1.05	.13
Father or others (reference)	-	-	-
**Education levels of the caregivers**			
Senior high school or below	0.66	0.58–0.75	< .001
College or above (reference)	-	-	-
**Number of people in the family**			
Two to four people	0.94	0.83–1.07	.36
Five people or above (reference)	-	-	-

“Receive research feedback (telephone)”	aOR	95% CI	P value
**Areas of the participants**			
Urban	0.76	0.65–0.88	< .001

Suburban (reference)	-	-	-
**Age of the children, year**	1.02	0.95–1.09	.66
**Sex of the children**			
Female	1.13	1.00–1.29	.05
Male (reference)	-	-	-
**Age of the caregivers, year**	0.98	0.97–0.99	< .001
**Role of the caregivers**			
Mother	1.00	0.83–1.20	.97
Father or others (reference)	-	-	-
**Education levels of the caregivers**			
Senior high school or below	1.67	1.47–1.90	< .001
College or above (reference)	-	-	-
**Number of people in the family**			
Two to four people	0.88	0.77–0.99	.04
Five people or above (reference)	-	-	-
